# Quality-of-Life Endpoints in Women of Childbearing Age with Hidradenitis Suppurativa: A Tertiary-Care-Centre-Based Study

**DOI:** 10.31138/mjr.220823.qoe

**Published:** 2023-08-22

**Authors:** Aikaterini Tsentemeidou, Elena Sotiriou, Katerina Bakirtzi, Ilias Papadimitriou, Themis Chatzi-Sotiriou, Angeliki Panagopoulou, Nikolaos Kougkas, Aimilios Lallas, Efstratios Vakirlis

**Affiliations:** 1 First Department of Dermatology and Venereology, School of Medicine, Aristotle University, Thessaloniki, Greece,; 2 Department of International and European Studies, School of Social Sciences, Humanities and Arts, University of Macedonia, Thessaloniki, Greece,; 3 Fourth Department of Internal Medicine, Aristotle University of Thessaloniki, Greece

**Keywords:** hidradenitis suppurativa, women, female, quality of life, childbearing age, pregnancy

## Abstract

**Background:**

Hidradenitis suppurativa (HS) principally affects women of childbearing age, who face gender-specific challenges and have lower life-quality than men. HS also seems to impact desire for procreation.

**Objective:**

To investigate various quality-of-life endpoints in women of childbearing age with HS.

**Study design:**

A cross-sectional questionnaire-based study was performed at a university dermatology department. Eighteen yes/no and one open-ended questions explored impact of HS on social life, sexual life, family planning, working life and healthcare-backed support. A sensitivity analysis was performed for women under 25, who are significantly less likely to be married/in a permanent relationship in Greece, as this could act as a confounding factor regarding family planning.

**Results:**

Ninety-six women were included. Most women (80.8%) carry a stigma because of HS, which also affects their choice of clothes and social relationships. Sexual impairment affects 73.1% of women. One third of women wants less or no children because of HS, 67.7% worry about its impact on pregnancy, birth, and the postpartum, and 84.6% worry about the impact of HS treatment on fertility and their babies’ health. Almost 43% fear losing their job because of HS, 34.4% are discriminated against at work and 33.3% state HS has hindered their career. Most women are not adequately informed about their disease or available support groups/material and 41.7% have not received good enough care through pregnancy/postpartum.

**Conclusions:**

Life-quality endpoints should be meticulously screened in women. Multidisciplinary-led treatment should be offered during pregnancy and the postpartum.

## INTRODUCTION

Hidradenitis suppurativa (HS) is a chronic autoinflammatory skin disease, which manifests itself with painful malodorous inflammatory nodules and abscesses, fistulas, and scars located to intertriginous skin.^1^ Its severely negative impact on quality-of-life, comparable to this of serious systemic diseases, has been well documented; notably, HS patients would give up more years of their life, to live free of their skin disease than free of obesity.^1–3^ A major component of reduced life quality is sexual health impairment, which is significantly more frequent among HS patients than among healthy controls.^4–6^ A multinational study examining 3,485 subjects with more than twenty dermatological disorders found that sexual impairment was most common among HS patients (66.7%).^7^ HS impact on work productivity, both in the form of absenteeism and presenteeism, is a further crucial implication of this disease.^8^

HS principally affects women of childbearing age.^2,4,9–11^ Aside from the detrimental disease symptoms and sequalae common to both men and women, the latter also face difficulties germane to their gender, such as menstruation-related flares, adverse pregnancy outcomes, limitations regarding treatment during pregnancy and the postpartum, birth and breastfeeding challenges, issues with pelvic examination, as well as breast examination and imaging tests.^2,10,12–16^ What is more, women seem to fare worse on quality-of-life endpoints comparing to their male counterparts: their overall sexual impairment is greater, they are more likely to have body image issues, have greater fear of rejection when it comes to relationships, and manifest significantly more symptoms of depression.^5,17–20^

The impact of HS is felt by patients’ families as well.^2^ For many of the above reasons, as well as because of patients’ fear of vertically transmitting the disease to their offspring, HS can potentially influence desire for procreation.^3,20–23^ Indeed, approximately half of asked women with HS (50.96%, 53/104) did not fulfil their reproductive desire according to a prospective observational study.^21^ Research has meanwhile revealed that clinician-led counselling regarding family planning is lacking, which in turn reflects the paucity of relevant practice guidelines.^22^ Given the seemingly greater effect of HS on women, it is vital that we gain a better insight into how the disease impacts women’s lives, elucidate the unique challenges they face and establish guidelines and support mechanisms for their handling. To this end we performed a questionnaire-based study aimed at women with HS, focusing on various quality-of-life endpoints.

## MATERIALS AND METHODS

A cross-sectional questionnaire-based study was performed according to Strengthening The Reporting of OBservational studies in Epidemiology (STROBE) statement. Candidate subjects were sourced from the First Department of Dermatology and Venereology, Aristotle University, Thessaloniki, Greece, a tertiary healthcare unit, from January 1^st^ until August 31st, 2022. Recruitment and data collection were performed from January 1^st^ until September 30th, 2022. All adult women of childbearing age (18-49 years old according to World Health Organization), who visited the department and had a formal diagnosis of HS with symptom onset at least 6 months before, were considered eligible for inclusion. Women who had entered menopause were excluded. Examination, selection, and recruitment of subjects were performed by a consultant dermatologist with special interest in HS (ES). All participating subjects signed informed consent forms.

The questionnaire was developed by two investigators (AT and ES) based on existing literature in this patient population and was then piloted with five patients willing to provide feedback. It included eighteen questions with yes, no, cannot recall/haven’t thought about it, not applicable as potential answers, and one open-ended question, regarding the impact of HS on social life, sexual life, family planning, working life, and satisfaction with healthcare-backed support (**Table 1**). The questions were asked and explained by the recruiting investigator (ES). Demographic characteristics, time of symptom-onset, current treatment, history of treatment with biologics, Hurley stage and Physician Global Assessment of disease activity (PGA) on a scale of 0-5 (clear skin, almost clear skin, mild disease, moderate-to-severe disease, severe disease) were also documented. Family-planning questions could be answered retrospectively as well as prospectively (potential recall bias was minimised through carefully structured questions, allowing sufficient time for recall, and cross-referencing with family members, if present).

**Table 1.** Study questionnaire.

**Eligibility criteria**	Woman, 18–49 years old
	Has not entered menopause

**Demographic characteristics**	Date of birth, date of symptom onset, current treatment, history of biologic treatment

**Clinical examination**	PGA^a^, Hurley stage

**Social life**	1. I feel stigmatised because of my disease
	2. I try to hide my disease, and this affects how I pick my clothes
	3. My disease affects my social life and friendships

**Sexual life**	4. My sexual life is impaired because of my disease
	5. I feel embarrassed when removing my clothes during sexual intercourse/I don’t want to remove them
	6. I have had/currently have a partner who feels uncomfortable with my skin lesions or is afraid that my disease is contagious, and therefore avoids sexual intercourse

**Family planning**	7. I have decided/am seriously thinking of not having children or having less children because of my disease
	8. I am afraid that my disease will have an impact on my children’s health and/or my ability to take care of them
	9. I am afraid that pregnancy/the postpartum will have a negative impact on my disease
	10. I am afraid that the medication I am taking will have an impact on my fertility or will harm my baby during pregnancy or lactation
	11. I have faced problems with pregnancy, birth, postpartum or breastfeeding because of my disease (what kind of problems?)

**Working life**	12. I am afraid I will lose my job because of my disease
	13. I have been absent from work/less productive at work because of my disease
	14. How many days sick leave have I taken due to my disease during the past year
	15. Have I been discriminated against at work because of my disease
	16. My disease has a negative impact on my income or my career

**Healthcare provider- backed support**	17. I am not adequately informed about my disease
	18. I have not received adequate support with/information about how to handle my disease during pregnancy and the postpartum
	19. I am not adequately informed about services/support groups/support material for people with hidradenitis suppurativa

Sample size was calculated through the following formula: N={Za^2^*[p*(1−p)]}/d^2^, where p is the prevalence of the studied endpoint, d the estimate precision, and Za is 1.96. Z score is a value that measures the difference of any given data point from the average of its data set in standard deviations. The value Z score receives for a 95% confidence level is 1.96, which means that there is a 2.5% area on the left and right side of the normal distribution curve acting as a rejection zone of the null hypothesis (women with hidradenitis have the same quality of life as healthy women).

Based on a multitude of existing data, the life quality of the majority of HS patients is severely affected, so we conservatively estimated that at least half of asked patients would answer yes (p=50). The required sample size was 96. All quality-of-life endpoints were dichotomous variables and were described through percentages. Normality of distribution was tested through Shapiro-Wilk test. Multivariate logistic regression with 95% Confidence Interval (two-tailed significance level of 0.05) was performed to check the impact of disease severity (Hurley stages II and III versus stage I), biologic treatment and disease duration on life-quality endpoints. Missing data was handled through case mean substitution.^24^ A sensitivity analysis was performed for women under 25, who are significantly less likely to be married/in a permanent relationship in Greece, as this could act as a confounding factor regarding family planning (participants may have not had the need to give the matter any thought and answered “no” as a result). All analyses were performed with SPSS Statistics 28.0 (Armonk, NY: IBM Corp).

This study was performed based on the Declaration of Helsinki and was approved by the Ethics Research Committee of the Hospital of Venereal and Cutaneous Diseases of Thessaloniki, Greece (53/02-12-2021).

## RESULTS

**Figure 1.**
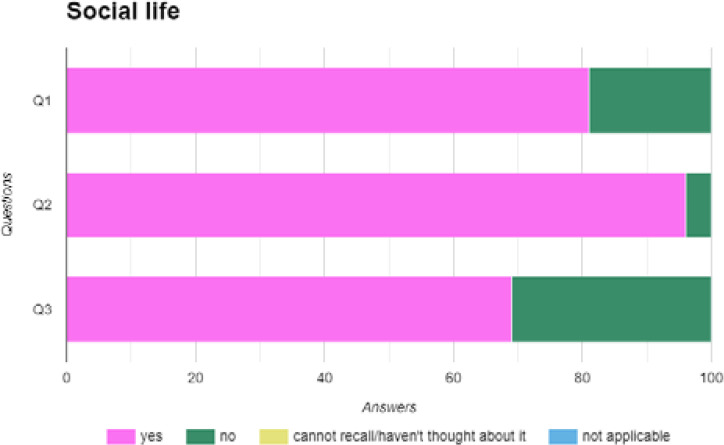
Q1 I feel stigmatized because of my disease, Q2 I try to hide my disease and this affects how I pick my clothes, Q3 My disease affects my social life and friendships; x axis: percentages.

**Figure 2.**
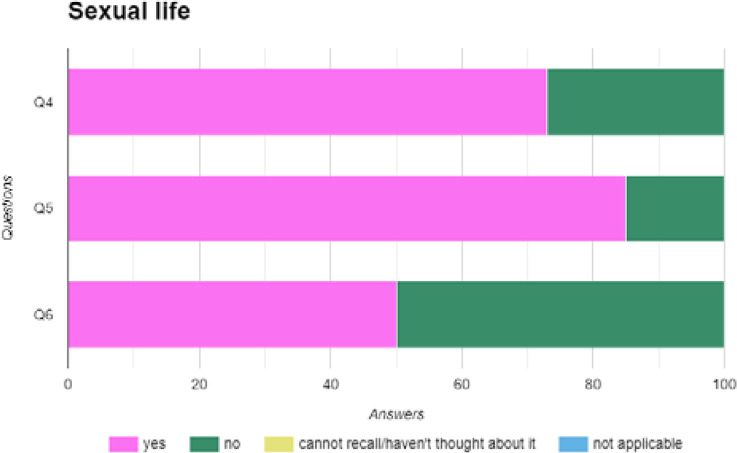
Q4 My sexual life is impaired because of my disease, Q5 I feel embarrassed when removing my clothes during sexual intercourse/I don’t want to remove them, Q6 I have had/currently have a partner who feels uncomfortable with my skin lesions or is afraid that my disease is contagious, and therefore avoids sexual intercourse; x axis: percentages.

**Figure 3.**
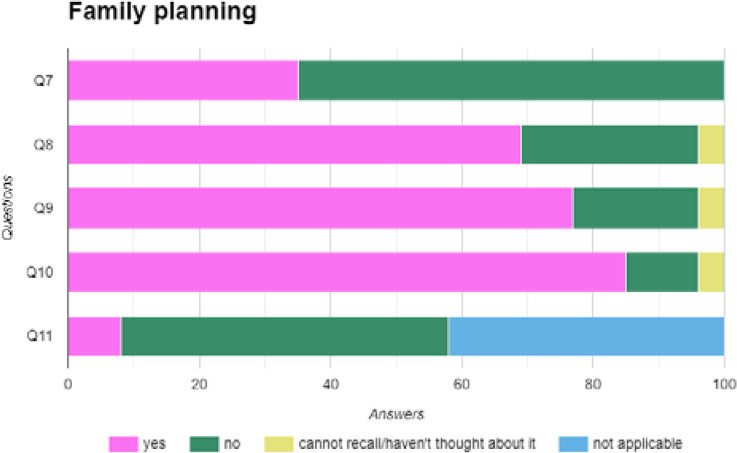
Q7 I have decided/am seriously thinking of not having children or having less children because of my disease, Q8 I am afraid that my disease will have an impact on my children’s health and/or my ability to take care of them, Q9 I am afraid that pregnancy/the postpartum will have a negative impact on my disease, Q10 I am afraid that the medication I am taking will have an impact on my fertility or will harm my baby during pregnancy or lactation, Q11 I have faced problems with pregnancy, birth, postpartum or breastfeeding because of my disease (what kind of problems?); x axis: percentages.

**Figure 4.**
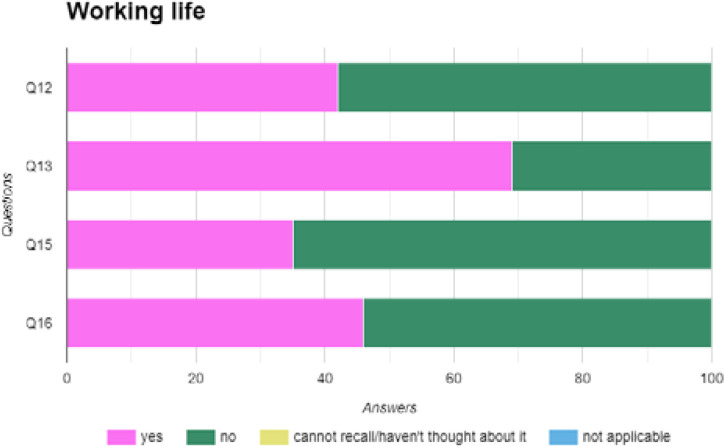
Q12 I am afraid I will lose my job because of my disease, Q13 I have been absent from work/less productive at work because of my disease, Q14 How many days sick leave have I taken due to my disease during the past year, Q15 Have I been discriminated against at work because of my disease, Q16 My disease has a negative impact on my income or my career; x axis: percentages.

**Figure 5.**
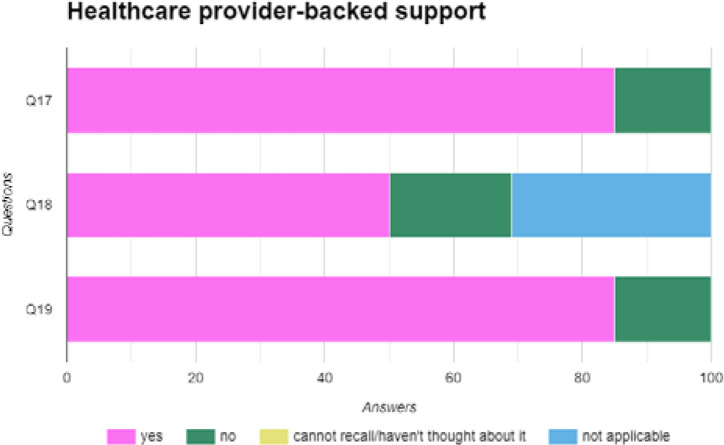
Q17 I am not adequately informed about my disease, Q18 I have not received adequate support with/information about how to handle my disease during pregnancy and the postpartum, Q19 I am not adequately informed about services/support groups/support material for people with hidradenitis suppurativa; x axis: percentages.

One hundred and twenty-two women with HS were screened, 97 of them (79.5%) were eligible for inclusion and 96 (78.6%) consented to participating in the study. Characteristics of study participants are presented in **Table 2**. Most patients had Hurley stage II (44.8%) and PGA 3 (40.6%) disease. Almost 39% of subjects were under biologic treatment and an equal number of them were under doxycycline. As questionnaires were answered with the help of and filled in by a consultant dermatologist (ES), there were no missing data. Questionnaire results are presented in **Figures 1–5**.

**Table 2.** Demographic characteristics.

**Age** (years, mean, 95% CI^a^)	34.95 (33.28, 36.62)	
**Disease duration** (years, mean, 95% CI)	12.14 (10.53, 13.75)	
**Current treatment** (%)	No treatment	2.1
	Topical only	20.8
	Systemic antibiotic(s)	38.5
	Biologic	38.5
**History of biologic treatment** (%)		40.6
**Hurley stage** (%)	Stage I	29.2
	Stage II	44.8
	Stage III	26.0
**Physician’s Global Assessment** (%)	Clear skin	0
	Almost clear skin	0
	Mild disease	34.4
	Moderate disease	40.6
	Moderate-to-severe disease	19.8
	Severe disease	5.2

### Social life

The great majority of women (80.8%) admitted to carrying a stigma because of HS. An even greater proportion of them (96.2%) stated that their disease interferes with how they dress: they pick the outfit that best hides their lesions, avoid “valuable clothes”, which could be stained by discharge, or tight clothing, which worsens their symptoms as a result of friction. Comparatively less participants (69.2%) declared that HS keeps them from having a satisfactory social life or making friends.

### Sexual life

The majority of asked patients (73.1%) declared that their sexual life is impaired as a result of HS. When asked in what way, popular answers were “not having sex as often as I would like”, “not feeling attractive”, “my partner is hesitant when I have flares”. Interestingly enough, even women who wouldn’t describe their sexual life as “impaired” (84.6%) conceded to not feeling comfortable removing their clothes during intercourse, as they are afraid that their “unsightly” lesions will affect their partner’s sexual desire. Half of asked women (50%) have had at least one partner avoid sexual intercourse because of their skin disease: they were either “discouraged” by the patient’s appearance or afraid of “catching” HS themselves.

### Family planning

More than one third of asked women (34.6%) reported that HS has influenced their desire for procreation, making them not want any children at all or want less children than they would, had they had disease-free skin. Most women (67.7%), including those who haven’t altered their family planning because of HS, admitted to entertaining worrying thoughts about if and how HS could affect their children’s wellbeing and/or their own ability to properly take care of their children, such as having a miscarriage or complications during pregnancy, having children with health issues related or unrelated to their skin, etc. On the other hand, most women (74%) fear that having children will worsen their disease and feel uncertain about being able to handle this additional burden. Medication received for the treatment of HS seems to cause worry in 84.6% of asked women, with regard to infertility and congenital malformations or other issues with pregnancy and the postpartum. A little less than half of study participants (42.3%) have not been pregnant; these were significantly younger than their counterparts (mean age 32.34 years, 95%CI 29.45, 35.23, p=0.07), which means that their age could partly explain their nulliparity. Of those who have been pregnant and/or had children (mean age 36.89 years, 95%CI 35.15, 38.71) 4.9% reported having had problems with breastfeeding (pain) and 1.9% reported having had a caesarean section because of HS lesions in the genital area.

### Working life

A little less than half of asked women (42.7%) declared fear of losing their job because of their disease. This was most often due to frequent sickness leave and/or reduced productivity at work as a result of pain, self-consciousness or poor mental health. The majority of women (68.8%) did indeed report having been absent from work (absenteeism) or less productive (presenteeism) at work during disease flares, with a median 7 days (interquartile range 10) of sickness leave due to HS during the preceding year. The minority of asked women (34.4%) stated that they have faced discrimination at work because of their disease mostly in the following form: “colleagues believe I just try to skip work or that I am lazy, because they can’t actually see my lesions and don’t understand how bad I feel”. Similarly, 33.3% feel that HS has had a negative impact on their income and career, as absenteeism and presenteeism have made them less competitive and consequently less likely candidates for promotion.

### Healthcare provider-backed support

The great majority of participants (82.3%) are not adequately informed about their disease, mainly in terms of its cause and behaviour, prognosis, comorbidities, and what to expect from treatment. A significant 41.7% stated that they haven’t received enough support through the challenging periods of pregnancy and the postpartum by their treating physician, including closer clinical supervision, reviewing of the treatment plan, preparing for birth and the postpartum, and collaborating with the obstetrics team. Finally, 79.2% feel they are not adequately informed about support groups, services, and material dedicated to helping patients with HS; they almost unanimously believe that providing access to these amenities should be part of the care they receive from their treating physician.

### Factors influencing quality-of-life endpoints

Regarding most quality-of-life endpoints (questions Q1 & Q3 of social life, Q4-Q6 of sexual life, Q8-Q10 of family planning, Q12-Q15 of working life, Q18 & Q19 of healthcare support) having Hurley stage II and III disease, as opposed to stage I, made it statistically significantly likelier to answer “yes” (be burdened). History of treatment with biologic agents was also a predictive factor for answering “yes” to Q9 of family planning, Q16 of working life and Q18 of healthcare support. Since biologic agents are usually stopped when pregnancy is confirmed or before the third trimester, being afraid that pregnancy will lead to a flare of the disease or not receiving enough information/support/education regarding the use of biologics during pregnancy and the postpartum is not unexpected. Disease duration made it likelier to answer “yes” to Q19 of healthcare support (information about support groups and services), as the longer a patient has a disease, the likelier they are to expect guidance with support resources. Table 3 shows Odds ratio values and statistical significance.

**Table 3.** Factors significantly increasing likelihood of answering “yes”.

**Question**	**Factor**	**Odds ratio**	**Sig. level**	**95%CI**
Q1	Hurley stage	125.035	p<0.001	9.775, 1599.331
Q2	None	-	-	-
Q3	Hurley stage	33.860	p<0.001	8.190, 139.984
Q4	Hurley stage	44.135	p<0.001	10.661, 182.717
Q5	Hurley stage	44.320	p<0.001	7.079–277.480
Q6	Hurley stage	16.304	p<0.01	4.449–59.746
Q7	None	-	-	-
Q8	Hurley stage	8.203	p<0.01	2.711–24.821
Q9	Hurley stage	3.935	p=0.019	1.255–12.341
	Biologic treatment	4.997	p=0.025	1.229-20.317
Q10	Hurley stage	6.082	p=0.08	1.595–23.200
Q11	None	-	-	-
Q12	Hurley stage	5.195	p=0.09	1.500–17.990
Q13	Hurley stage	9.565	p<0.001	3.106–29.456
Q15	Hurley stage	4.538	p=0.03	1.157–17.797
Q16	Biologic treatment	3.254	p=0.019	1.218–8.694
Q17	None	-	-	-
Q18	Biologic treatment	3.382	p=0.05	1.440–7.944
Q19	Hurley stage	5.960	p=0.02	1.900–18.695
	Disease duration	0.928	p=0.031	0.867–0.993
	**Factor**	**B**	**Sig. level**	**R**
Q14	Hurley stage	5.300	p<0.001	0.463

### Sensitivity analysis

**Figure 6.**
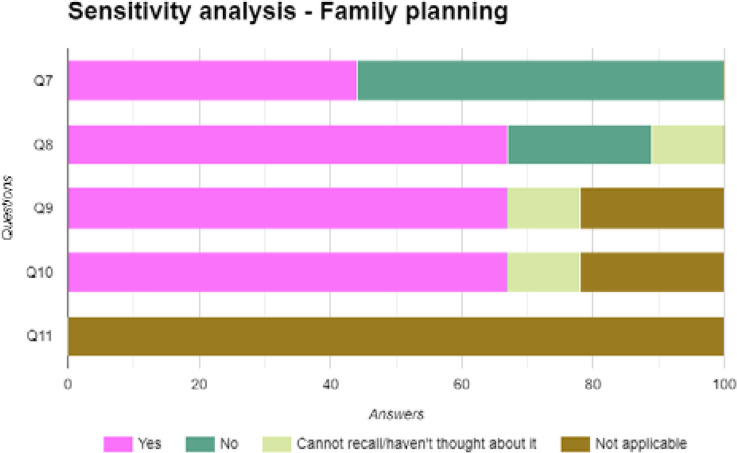
Q7 I have decided/am seriously thinking of not having children or having less children because of my disease, Q8 I am afraid that my disease will have an impact on my children’s health and/or my ability to take care of them, Q9 I am afraid that pregnancy/the postpartum will have a negative impact on my disease, Q10 I am afraid that the medication I am taking will have an impact on my fertility or will harm my baby during pregnancy or lactation, Q11 I have faced problems with pregnancy, birth, postpartum or breastfeeding because of my disease (what kind of problems?); x axis: percentages.

Women under 25 years of age were statistically significantly less likely to answer “yes” to questions 8-11 of family planning (Q8 p=0.007, Q9 p<0.001, Q10 p<0.001, Q11 p=0.001) comparing to their older counterparts (**Figure 6**) and none of them had been pregnant before the study was conducted. A possible explanation for this observation is that due to their young age, these women were not likely or willing to have children in the near future, thus not being really concerned with issues related to pregnancy and the postpartum.

## DISCUSSION

According to this questionnaire-based study, the majority of female patients with HS aged between 18 and 49 years declare themselves burdened in almost all key areas of life quality: they feel socially stigmatised, their sexual life is impaired, they worry about how HS may influence pregnancy, the postpartum and their children’s health, their working life is negatively impacted and, last but not least, they do not believe they have received enough support and information from healthcare professionals. Hurley stages III and II are significantly associated with reduced life-quality in most fields. Women under 25 are significantly less likely to be concerned about problems related to pregnancy and the postpartum or to have experienced such problems. Limitations of this study are the relatively small sample size, the lack of a control group, providing only yes/no as possible answers without the potential for response grading (for example on a scale of 0 to 10 or with the help of a visual analogue scale).

HS has immense psychosocial impact, as it is a long-lasting painful disease with malodour and disfiguring scars.^25^ Stigmatization and clothing limitations are customary among HS patients and are more frequent and severe than that experienced as a result of other skin diseases.^26,27^ Our study revealed that the great majority of women with HS carry a stigma and feel restricted in their choice of clothing. What is more, most of them stated that their ability to make friends and their social life on the whole are impaired because of HS.

The majority of women with HS have sexual impairment, measured with validated tools; reportedly, although both sexes are affected, women score worse than men.^3^ This is possibly attributed to women’s different psychosocial role and increased sensitivity to their external environment.^3^ Our results agree with existing data, as sexual impairment was prevalent in most women.

A meta-analysis synthesising data regarding the impact of HS on pregnancy has shown that spontaneous abortion and gestational diabetes happen more often in HS patients comparing to healthy controls.^28^ HS seems to impinge on delivery-method as well, as caesarean section has been chosen over vaginal delivery in cases of severe anogenital involvement.^16,28^ Formation of new lesions or trouble healing have been reported on section scar.^12,13,16,29^ What is more, lesions on the breasts can hinder lactation, which is why they should be treated timely.^11,12^ There is also evidence that women with HS fear their child will have the disease, or delivery will be more difficult because of it.^22^ Our study showed that most women worry about potential difficulties during pregnancy and the postpartum, including whether they will be fit enough to properly take care of their children. Almost half of them have experienced problems, mainly in relation to breastfeeding.

HS has a profound impact on patients’ working life. HS patients are more frequently unemployed, have slower annual income growth and higher risk of stopping work than healthy controls (p<0.05) and longer annual sickness leave (p<0.001).^30,31^ Absenteeism has been reported among 50-58.1% of HS patients, with a mean number of sick days of 14.2-33.6% (range 2-120) per year across various studies.^30^ Almost a quarter of asked HS patients (23.3%) reported hindered career advancement as a result of their disease.^30^ Our study confirms this data.

According to a 2020 survey, 77% of HS patients participate in support groups, mostly over the internet (89%).^32^ Patients in our study expressed their disappointment in the amount and quality of information, education, and referral to support groups they have received from health-care professionals, especially during pregnancy and the postpartum. Similar concerns have been documented before: HS patients have stated that their physician had not satisfyingly tackled their sexual impairment issues and would like doctors to devote more time to their sexual health.^33^ On the other hand, healthcare providers with greater experience (HS specialty directors) feel more comfortable handling pregnant patients with HS.^34^

Taking gender differences into account when treating patients has been gaining ground over recent years.^9^ Female patients with HS should be carefully examined for reduced quality of life, which should guide treatment decisions alongside clinical severity. Education, support, and carefully devised treatment plans, which take the patient’s wishes into consideration, should be offered to women who are or planning to get pregnant. Multidisciplinary teams involving dermatologists, obstetricians/gynaecologists, psychiatrists and/or psychologists and endocrinologists should be devised for optimal care, especially for the challenging periods of pregnancy and the postpartum. Well-designed studies are needed to assess the safety of existing and new drugs for HS during pregnancy and lactation.

## Abbreviations:

HS: Hidradenitis Suppurativa;
PGA: Physician Global Assessment of disease activity;
STROBE: Strengthening The Reporting of OBservational studies in Epidemiology
